# Microvesicles Derived from Human Embryonic Neural Stem Cells Inhibit the Apoptosis of HL-1 Cardiomyocytes by Promoting Autophagy and Regulating AKT and mTOR via Transporting HSP-70

**DOI:** 10.1155/2019/6452684

**Published:** 2019-10-24

**Authors:** Lei Zhang, Jianyi Gao, Tianyan Chen, Xiang Chen, Xianyan Ji, Kai Ye, Jiahong Yu, Bin Tang, Yusheng Wei, Hong Xu, Jiabo Hu

**Affiliations:** ^1^Jiangsu Key Laboratory of Medical Science and Laboratory Medicine, School of Medicine, Jiangsu University, 301 Xuefu Road, Zhenjiang, Jiangsu 212013, China; ^2^Translational Medicine Center, Department of Laboratory Medicine, The Affiliated Wuxi No. 2 People's Hospital of Nanjing Medical University, Wuxi, Jiangsu Province 214002, China; ^3^Department of Clinical Laboratory, The Second Affiliated Hospital of Nantong University, 6 Haierxiang North Road, Nantong, Jiangsu 226001, China; ^4^Department of Clinical Laboratory, Zhenjiang Centre for Disease Prevention and Control, 9 Huangshan South Road, Zhenjiang, Jiangsu 212013, China

## Abstract

Myocardial reperfusion injury (MRI) induced by cardiomyocyte apoptosis plays an important role in the pathogenesis of a variety of cardiovascular diseases. New MRI treatments involving stem cells are currently being developed because these cells may exert their therapeutic effects primarily through paracrine mechanisms. Microvesicles (MVs) are small extracellular vesicles that have become the key mediators of intercellular communication. MVs derived from stem cells have been reported to play an important role in MRI. In this article, we attempted to explore the mechanisms by which MVs derived from human embryonic neural stem cells (hESC-NSC-derived MVs) rescue MRI. hESCs were differentiated into NSCs, and MVs were isolated from their supernatants by ultracentrifugation. H_2_O_2_ was used to induce apoptosis in HL-1 cardiomyocytes. Cell viability was detected by using the CCK-8 assay, apoptosis was detected by Annexin V-FITC/PI staining, and apoptosis-related proteins and signalling pathway-related proteins were detected by western blot analysis. Autophagic flux was measured using the tandem fluorescent mRFG-GFP-LC3 assay. Transmission electron microscopy and western blot analysis were adopted to evaluate autophagy levels. hESC-NSC-derived MVs increased the autophagy and inhibited the apoptosis of HL-1 cells exposed to H_2_O_2_ for 3 h in a dose-dependent manner. Additionally, hESC-NSC-derived MVs contained high levels of heat shock protein 70 (HSP-70), which can increase the level of HSP-70 in cells. Moreover, the same effect could be achieved by heat shock preconditioning of HL-1 cells overexpressing HSP-70. The benefits of NSC-MVs may be due to the involvement of AKT and mTOR signalling pathways. Importantly, hESC-NSC-derived MVs stimulated the activation of the AKTand mTOR signalling pathway in those cells by transporting HSP-70. Our results suggest that hESC-NSC-derived MVs inhibit the apoptosis of HL-1 cardiomyocytes by promoting autophagy and regulating AKT and mTOR via transporting HSP-70. However, this hypothesis requires in vivo confirmation.

## 1. Introduction

Ischaemic heart disease (IHD) is one of the leading causes of death and disability worldwide [1]. Timely reperfusion is the main treatment for IHD, which not only reduces infarct size but also prevents heart failure. However, the reperfusion process itself can induce myocardial cell death, which is referred to as myocardial reperfusion injury [2]. Myocardial reperfusion injury is caused by reactive oxygen species overproduction [3], energy metabolism disorders, neutrophil infiltration, calcium overload, and vascular endothelial dysfunction, but there is still no effective treatment [4].

Cell therapy is considered a viable option for treating myocardial reperfusion injury. Stem cell transplantation is an effective method that primarily improves damaged tissues by releasing autocrine and paracrine factors. However, major concerns such as teratoma formation, immune responses, difficulty of harvesting cells, and limited cell proliferation and differentiation hinder the routine use of these cells as a treatment option in the clinic. The emergence of stem cell-derived extracellular vesicles (EVs; also known as exosomes and microvesicles) circumvents these problems while still providing growth factor miRNAs and other cytoprotective factors that help repair and regenerate damaged tissues [5].

EVs are bilayered lipid vesicles that are 100-1000 nm in diameter and are secreted by most types of cells [6]. EVs were originally thought to be a mechanism that cells use to remove unwanted cellular components [7] but are now recognized as natural carriers of many signalling molecules that mediate cell-cell communication, including lipids, proteins, DNA, mRNAs, miRNAs, siRNAs, and lncRNAs [8]. Once attached to a target cell, EVs can induce signalling via receptor-ligand interaction or can be internalized by endocytosis and/or phagocytosis or may even fuse with the target cell's membrane to deliver their content into its cytosol, thereby modifying the physiological state of the recipient cell [6]. EVs are nanosized vesicles that are stable, biocompatible, nonmutagenic, and biological barrier permeable and exhibit low immunogenicity [9]. Recent studies have demonstrated that EV-mediated crosstalk between different cell types in the heart plays an important role in maintaining cardiac homeostasis and the pathogenesis of heart disease [10]. Mouse embryonic stem cell-derived exosomes have been shown to enhance infarcted heart neovascularization and myocardial cell survival and reduce fibrosis after infarction [11]. Restoration of the miR-21 pathway using cardiac progenitor cell-derived exosomes can protect myocardial cells against oxidative stress-related apoptosis [12]. In addition, miR-126 and miR-130 were found to be significantly increased in exosomes isolated from haematopoietic stem cells, promoting infarcted cardiac angiogenesis [13]. Neural stem cells (NSCs) and their derived EVs play a significant role in improving cerebral ischaemia-reperfusion injury and can significantly improve neurological deficits and reduce the volume of cerebral infarction, while retaining mitochondrial ultrastructure. In addition, they can effectively reduce oxidative stress, inhibit cell apoptosis, and promote angiogenesis [14–16]. These effects are also important in the treatment of IHD.

In this study, we first found that MVs derived from human embryonic neural stem cells (hESC-NSC-derived MVs) contain high levels of HSP-70, which can effectively inhibit cardiomyocyte apoptosis, and we explored the underlying mechanism.

## 2. Materials and Methods

### 2.1. Ethics, Consent, and Permissions

This study was performed in accordance with the principles of the Helsinki Declaration and was approved by the Ethical Review Board of Jiangsu University.

### 2.2. Cell Culture and Derivation of NSCs from hESCs

NSC culture and derivation were previously described [17–19]. Briefly, we obtained the hESC line SHhES2 [20] (passage 49) from the stem cell bank of the Chinese Academy of Sciences under a Materials Transfer Agreement. The hESCs were cultured on Matrigel (Corning, USA) and grown in mTeSR™1 (STEMCELL Technologies, Canada). The hESCs were ready to passage when the majority of colonies were large and compact and had centres that were dense compared to their edges. Then, 1 ml of dispase solution (STEMCELL Technologies, Canada) was added per well of hESCs, followed by incubation for 5 min at 37°C. When the colony edges began to curl up, the dispase was aspirated, and the cells were gently washed three times with Dulbecco's modified Eagle's medium: Nutrient Mixture F-12 (DMEM/F12; Gibco, USA). A sterile 1 ml pipette was used to detach colonies by scraping. After the cells were detached, fresh mTeSR™1 medium was used to resuspend the cells, after which they were seeded on the prepared Matrigel-coated plates. When the hESCs reached 70-80% confluence, the cells were washed with DMEM/F12, and the mTeSR™1 medium was replaced with neural induction medium (DMEM/F12, 1×B27, 20 ng/ml basic fibroblast growth factor (bFGF), 20 ng/ml epidermal growth factor (EGF), 1% nonessential amino acids, and 1% penicillin streptomycin). The cells were again washed with DMEM/F12 after 18-21 days and then dissociated with Accutase (STEMCELL Technologies, Canada) for 5 min at 37°C. Finally, the cells were resuspended in neural induction medium and seeded on the prepared Matrigel-coated plates (human embryonic stem cell derived-neural stem cells, P0 hESC-NSCs). The medium was changed every 2-3 days.

Murine cardiomyocyte HL-1 cells, a gift from Professor Su (Department of Immunology, Jiangsu University, Zhenjiang, China), were cultured in high-glucose DMEM containing 10% fetal bovine serum (FBS; Gibco, USA) and 1% antibiotics (streptomycin and penicillin) at 37°C in humid air with 5% CO_2_ and were passaged using 0.25% trypsin.

### 2.3. MV Isolation

The MV isolation procedures were performed as previously described [21]. In brief, the supernatants (or conditioned medium) of hESC-NSCs (P8-P15) were centrifuged at 2000 ×g for 5 min and then at 12,000 ×g for 15 min to remove cell fragments and impurities. Thereafter, the conditioned medium was centrifuged (Beckman Coulter Optima L-90K, Beckman Coulter, Fullerton, CA, USA) at 100,000 ×g for 2 h, washed in PBS, and subjected to a second ultracentrifugation step under the same conditions. We subsequently removed the excess fluid and added 200 *μ*l of PBS to resuspend the pellets, followed by storage at −80°C. The protein content of the hESC-NSC-derived MVs was analysed by the bicinchoninic acid method (BCA; Sigma, USA).

### 2.4. Nanoparticle Trafficking Analysis

Analysis of the absolute size distribution of MVs was performed using the NanoSight NS300 system (NTA; Malvern, UK). By using NTA, particles are automatically tracked and sized based on Brownian motion and the diffusion coefficient. After isolation, the MVs were diluted in 1 ml of filtered PBS. Control medium and filtered PBS were used as controls. The detection threshold was similar in all the samples. Three recordings were performed for each sample.

### 2.5. Internalization of hESC-NSC-Derived MVs into HL-1 Cells

The hESC-NSC-derived MV labelling procedures were performed according to the protocols of the manufacturers. In brief, the hESC-NSC-derived MVs were labelled with Dil (Sigma, USA) for 15 min at 37°C in the dark and subsequently washed three times in PBS with centrifugation at 100,000 ×g at 4°C for 2 h. Then, the labelled MVs (20 *μ*g/ml) were added to the prepared HL-1 cells for 24 h. After incubation, the cells were washed once with PBS. Cells that received PBS were used as controls. Nuclear staining was performed using 4′,6-diamidino-2-phenylindole (DAPI; Thermo Fisher, USA). Cell images were obtained via Olympus FV1000 laser-scanning confocal microscopy (Olympus, Japan).

### 2.6. Sodium Dodecyl Sulfate-Polyacrylamide Gel Electrophoresis (SDS-PAGE) Analysis

Electrophoretic separation of MVs was performed on a Bio-Rad MiniProtean II system (Bio-Rad, USA). MVs were lysed in lysis buffer, and the lysis solution was mixed with SDS loading buffer (Bio-Rad, USA) containing 0.6 M *β*-mercaptoethanol (Bio-Rad, USA). Total protein was denatured by boiling for 10 min and immediately placed on ice. Protein loading in the gel was 20 *μ*g per lane. A 10% (*W*/*V*) polyacrylamide separating gel and a 4% (*W*/*V*) polyacrylamide stacking gel were used to resolve proteins at 120 V for 100 min. After separation, the proteins in the gel were stained with 0.1% Coomassie brilliant blue R250 (Sigma, USA), 10% acetic acid, and 40% ethanol for 1 h and then destained in 10% acetic acid and 40% ethanol.

### 2.7. Cell Viability Assay

The CCK-8 assay was used to measure HL-1 cell viability. HL-1 cells (1 × 10^5^ cells/well) were seeded in 96-well plates overnight. To detect the negative effects of H_2_O_2_ (Sigma, USA) on HL-1 cell viability, the cells were incubated with different concentrations (50, 100, 200, and 500 *μ*M) of H_2_O_2_ for 3 h; normal culture media were used for the control group. At the prespecified time points, 10 *μ*l of CCK-8 solution (MCE, USA) was added to the cells. After incubation for another 4 h, optical density (OD) values were determined at 450 nm using a microplate reader (BioTek, USA). Each group was tested in triplicate in three replicate wells. The cell viability or proliferation rate of the treated cells was calculated as a value relative to the control group. We calculated cell viability by using the following formula: cell viability = [(mean OD value of test groups)/(mean OD value of control groups)] × 100%.

### 2.8. Flow Cytometry Analysis

For these assays, 1 × 10^5^ cells were dissociated with Accutase, washed with PBS, fixed with 4% paraformaldehyde, and permeabilized with 5% FBS/PBS and 0.1% Triton X-100. The cells were then stained with fluorochrome-conjugated mouse anti-human antibodies (nestin and mouse IgG1 K Iso Control Alexa FluorTM488 Conjugate, eBioscience Thermo Fisher, USA) for 30 min on ice and washed with 5% FBS/PBS. Background staining for the antibodies was determined with matched fluorochrome-conjugated isotype controls. After immunostaining, the cells were resuspended in 0.3 ml of 5% FBS/PBS and analysed with a flow cytometer (FACSCalibur; BD Biosciences, USA).

The apoptosis of HL-1 cells was measured using an Annexin V-FITC/PI apoptosis kit (BD Biosciences, USA). In total, 1 × 10^5^ cells were collected and successively stained with 5 *μ*l of Annexin V-FITC and PI at normal temperature for 15 min away from light and were then analysed on a flow cytometer within an hour.

### 2.9. Immunofluorescence Staining

When cells reached 70-80% confluence, they were fixed with 4% paraformaldehyde overnight at 4°C. The cells were then blocked and permeabilized for 1 h at 37°C in PBS with 2% bovine serum albumin (BSA; Thermo Fisher, USA) and 0.1% Triton X-100, followed by incubation with rabbit anti-nestin (Boster, China), rabbit anti-pax6 (Boster, China), rabbit anti-GFAP (Boster, China), rabbit anti-S100 (Boster, China), and rabbit anti-*β*-III tubulin (Applied Biological Materials Inc., Canada) at the appropriate dilution overnight at 4°C. After washing with PBS, the cells were incubated at 37°C for 2 h with appropriate secondary antibodies: CyTM3-conjugated AffiniPure goat anti-rabbit IgG (H+L) (Jackson, USA). Nuclei were counterstained with DAPI. Images were captured using an Olympus inverted fluorescence microscope (IX73, Olympus, USA).

### 2.10. Western Blot Analysis

Briefly, HL-1 cells and MVs were lysed in lysis buffer, after which the total proteins of the cells and MVs were extracted. The protein was then subjected to SDS-PAGE and subsequently electroblotted onto PVDF membranes. The membranes were incubated with the primary antibodies overnight at 4°C, followed by incubation with the corresponding secondary antibodies for 1 h at room temperature. The resultant protein bands were detected using ECL (Millipore, USA) reagents. Primary antibodies, including mouse anti-*β*-actin (Santa Cruz Biotechnology, USA) and anti-HSP-70 (Santa Cruz Biotechnology, USA) and rabbit anti-Beclin-1, anti-P62, anti-LC3, anti-Bax, anti-Bcl-2, anti-caspase3, anti-CD63, anti-mTOR, anti-pmTOR, anti-AKT, and anti-pAkt, were purchased from Cell Signaling Technology (CST, USA). The primary antibodies were detected using goat anti-rabbit or goat anti-mouse secondary antibodies (Thermo Fisher Scientific, USA).

### 2.11. Characterization of Autophagosomes by Transmission Electron Microscopy (TEM)

HL-1 cells were first fixed with 3% glutaraldehyde plus 2% formaldehyde in 0.1 M sodium cacodylate buffer solution. Then, the cells were fixed with 2% osmium tetroxide and dehydrated with propylene oxide and ethanol. Thereafter, the cells were placed in embedding moulds saturated with propylene oxide and incubated in an oven at 60°C for 48 h. Finally, 70 nm thin sections were prepared and observed under a TEM (HITACHI-HT7700, Japan). A total of 3-5 images per section were examined per group in the three independent experiments.

### 2.12. Autophagy Flux Assay Using mRFP-GFP-LC3

HL-1 cells were transfected with the tandem fluorescent-mRFP-GFP-LC3-adenovirus, which expresses a specific marker of autophagosome formation to detect autophagy, according to the manufacturer's instructions. The GFP signal is quenched in a lysosomal environment; in contrast, the RFP signal is more stable in an acidic environment [22]. Therefore, autophagosomes are labelled yellow (green and red) or red. Cell images were obtained using Olympus FV1000 laser-scanning confocal microscopy (Olympus, Japan).

### 2.13. Experimental Groups

**Figure 1:**
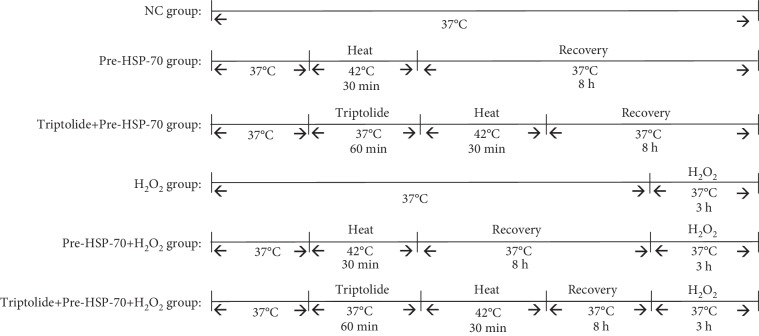
Experimental groups. NC group: the cells were kept in a 37°C in humid air with 5% CO_2_ incubator for the entire experiment. Pre-HSP-70 group: the cells were kept in a 42°C in humid air with 5% CO_2_ incubator for 30 min. Triptolide+pre-HSP-70 group: the cells were treated with triptolide 1 h before heat shock preconditioning. H_2_O_2_ group: the cells were kept in 1000 mM H_2_O_2_ for 3 h. Tripolide+H_2_O_2_ group: the cells were treated with triptolide 1 h before H_2_O_2_ treated. Pre-HSP-70+H_2_O_2_ group: 8 h after heat shock preconditioning, the cells were treated with H_2_O_2_ 3 h. Tripolide+Pre-HSP-70+H_2_O_2_ group: 1 h after triptolide treatment, cells were heat shock preconditioned for 8 h and then followed by treatment with H_2_O_2_ for 3 h after the Pre-HSP-70 treatment.

Heat shock preconditioning is used to generate HSP-70 and triptolide (APExBIO, USA) as an inhibitor of the heat shock response. Six groups of HL-1 cells were used in the present study (Figure 1): (1) NC group: cells were maintained in an incubator at 37°C in humid air with 5% CO_2_ for the entire experiment; (2) Pre-HSP-70 group: cells were maintained in an incubator at 42°C in humid air with 5% CO_2_ for 30 min; (3) triptolide+pre-HSP-70 group: cells were treated with triptolide for 1 h before heat shock preconditioning; (4) H_2_O_2_ group: cells were maintained in 1000 mM H_2_O_2_ for 3 h; (4) tripolide+H_2_O_2_ group: cells were treated with triptolide for 60 min before H_2_O_2_ treatment; (5) Pre-HSP-70+H_2_O_2_ group: 8 h after heat shock preconditioning, cells were treated with H_2_O_2_ for 3 h; and (6) tripolide+Pre-HSP-70+H_2_O_2_ group: 1 h after triptolide treatment, cells were heat shock preconditioned for 8 h and then followed by treatment with H_2_O_2_ for 3 h after the Pre-HSP-70 treatment.

### 2.14. Statistical Analyses

Data are expressed as the mean ± standard deviation (SD). Unpaired Student's *t*-tests and one-way ANOVA were used to compare the differences between groups. Each experiment was carried out independently at least three times. A *P* value of ≤0.05 was considered statistically significant.

## 3. Results

### 3.1. Characterization of hESC-NSC and hESC-NSC-Derived MVs

**Figure 2:**
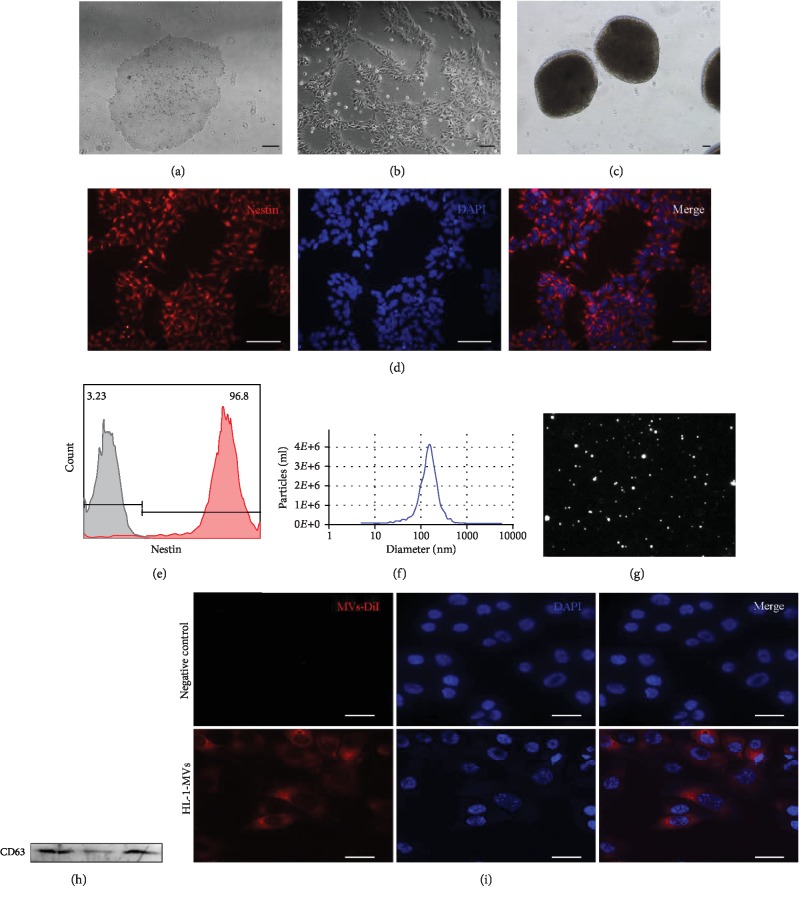
Characterization of hESC-NSC and hESC-NSC-derived MVs. The phase morphology of hESCs and NSCs growing on Matrigel-coated dish (a, b). Typical morphological neurospheres of NSCs (c). hESC-NSC were immunofluorescence staining for nestin (d). Flow cytometry analyzed purity of nestin, gray line: isotype control; red line: hESC-NSCs (e). Nanoparticle trafficking analyzed the diameters of MVs. The particles were 1.17*E* + 10 particles/ml, and the diameters of the particles were within the range of 50–1000 nm, with the average of 152.5 nm (f). A representative screenshot of the NTA video, the bright white dot indicates one moving particle (g). Western blotting characterized microvesicle marker CD63, three replicate samples (h). Representative confocal microscopy of HL-1 cells that was exposed to Dil-labelled MVs (i). ((a–d): scale bars 100 *μ*m; (h): scale bars 15 *μ*m).

The hESCs cultured under the feeder-free conditions grew into large compact colonies with clear and smooth edges (Figure 2(a)). Approximately 18 days after culture in neural induction medium, the cells began to take on an oval or, in some cases, triangular shape; the colonies showed a rosette-like structure, and most of them presented bi- or tripolar shapes (Figure 2(b)). A typical morphological neurosphere of the NSCs could form under suspension culture conditions (Figure 2(c)). Immunofluorescence staining revealed that the hESC-NSCs expressed nestin and pax6 (Figure 2(d), [Supplementary-material supplementary-material-1]). The hESC-NSCs we get could differentiate directly into glial cells and neurons ([Supplementary-material supplementary-material-1]). Additionally, the percentage of hESC-NSCs was determined through flow cytometry, and the results showed that up to 96.70 ± 0.70 of the population consisted of NSCs (Figure 2(e)). To obtain hESC-NSC-derived MV particles, the culture medium of hESC-NSCs was collected and precipitated. Then, the morphology and phenotypes of isolated particles were identified according to the characteristics of EVs described previously. The concentration and size range of the particles were measured through nanoparticle tracking analysis. The results demonstrated that the concentration of the particles was 1.17*E* + 10 particles/ml and that the diameters of the particles were within the range of 50-1000 nm, with an average of 152.5 nm (Figure 2(f)). Finally, the protein levels of microvesicle marker CD63 could be detected in the hESC-NSC-derived MVs (Figure 1(h)). Most Dil-labelled hESC-NSC-derived MVs can be internalized into the cytoplasm by HL-1 cells (Figure 2(i)).

### 3.2. hESC-NSC-Derived MVs Reduced Cell Apoptosis after H_2_O_2_ Stimulation

**Figure 3:**
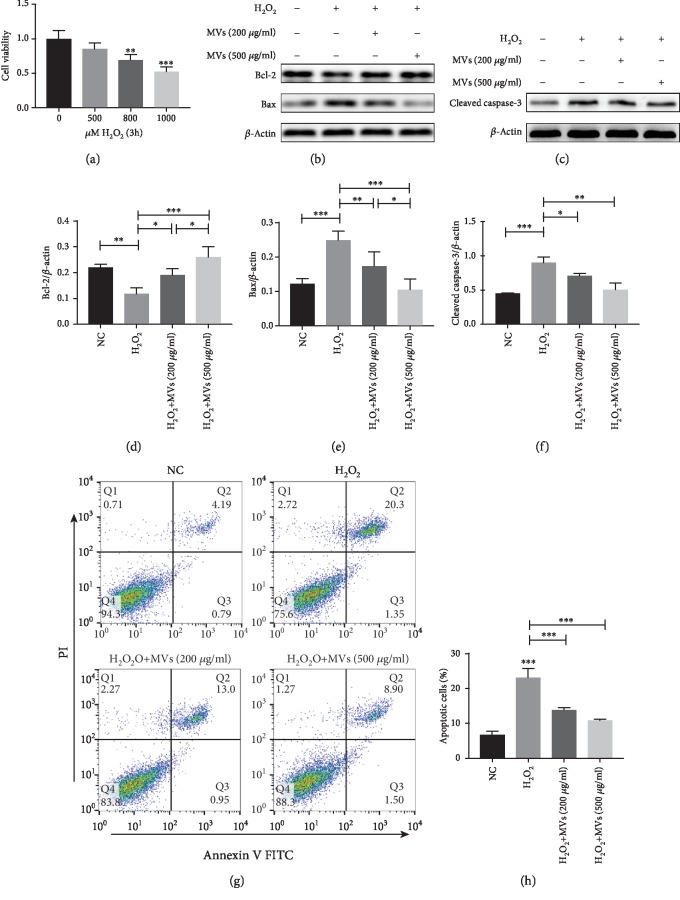
hESC-NSC-derived MVs reduced cell apoptosis after H_2_O_2_ stimulation. CCK-8 was used to measure HL-1 cell viability after exposure to 0, 500, 800, and 1000 *μ*M H_2_O_2_ for 3 h (a). Representative western blot images showing the protein levels of Bcl-2, Bax, and cleaved caspase-3. *β*-Actin was used as an internal control (b–f). Representative dot plots of cell apoptosis were showed after Annexin V/PI dual staining (g). The percentage of apoptotic cells was represent for both early and late apoptotic cells (h). Every experiment was repeated at least three times; error bars indicate mean ± SD (^∗^*P* < 0.05; ^∗∗^*P* < 0.01; ^∗∗∗^*P* < 0.001).

H_2_O_2_-stimulated HL-1 cells were used to mimic MRI. Compared with cells in the control group, H_2_O_2_ reduced cell viability, and viability was reduced to 50% at 3 h by the treatment of HL-1 cells with 1000 *μ*M H_2_O_2_ (Figure 3(a)). This concentration was therefore chosen for subsequent experiments. To determine the effects of MVs on HL-1 cell apoptosis induced by oxidative stress, we pretreated HL-1 cells with PBS or MVs for 24 h and then subjected them to acute H_2_O_2_ treatment, followed by the determination of apoptotic rates using flow cytometry. As shown in (Figures 3(g) and 3(h)), both 200 *μ*g of MVs and 500 *μ*g of MVs decreased the apoptosis rate of H_2_O_2_-treated HL-1 cells, but 500 *μ*g of MVs exerted a more pronounced effect. Western blotting was conducted to detect the ratio of Bcl-2, Bax, and cleaved caspase-3, which is a common way to assess the level of apoptosis. Similar results were obtained regarding the levels of Bcl-2, Bax, and cleaved caspase-3 in the control and other groups (Figures 3(b)–3(f)).

### 3.3. hESC-NSC-Derived MVs Increased the Level of Cell Autophagy after H_2_O_2_ Stimulation

**Figure 4:**
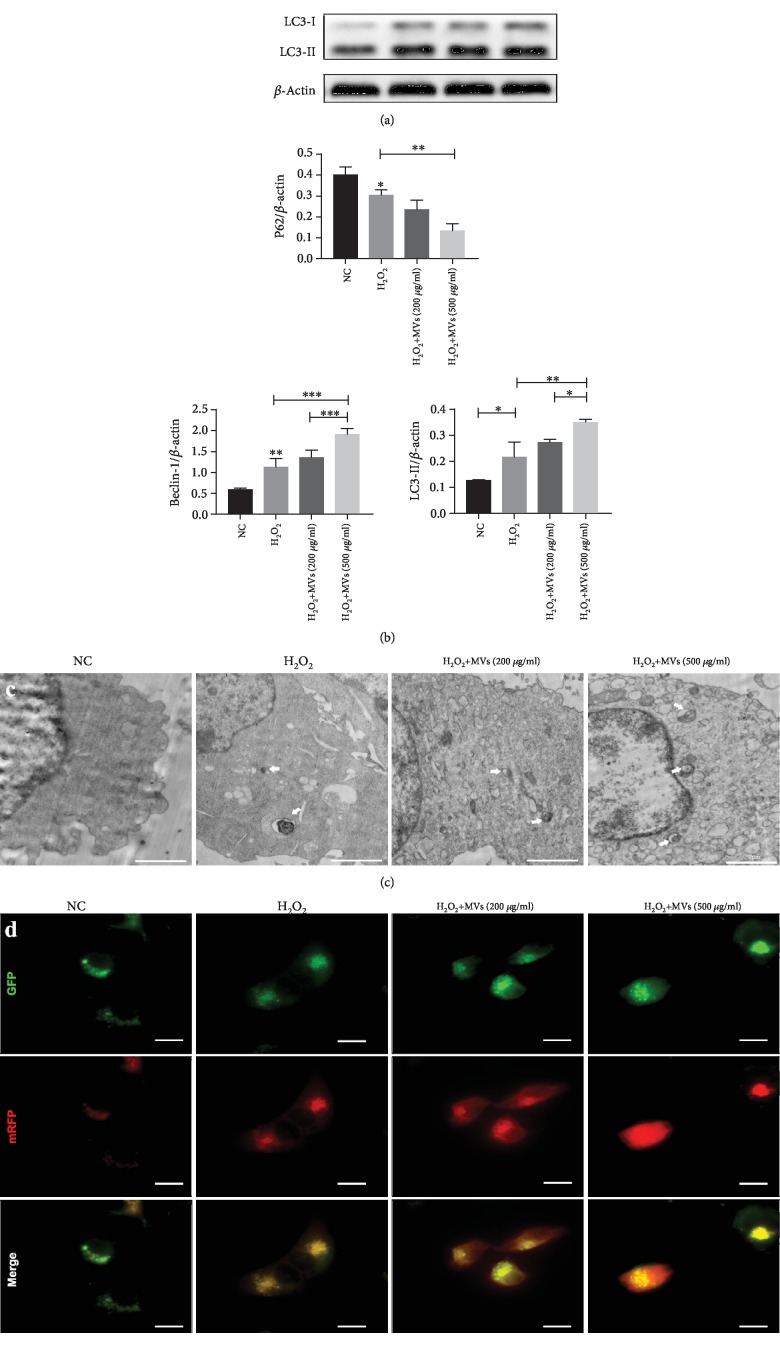
hESC-NSC-derived MVs increased the level of cell autophagy after H_2_O_2_ stimulation. Representative western blot images showing the protein levels of LC3, Beclin-1, and P62. *β*-Actin was used as an internal control (a, b). Autophagosomes were detected by tandem fluorescent mRFG-GFP-LC3 assay, scale bars: 15 *μ*m (c). Autophagosomes (white arrow) were measured by TEM in four groups, scale bars: 2 *μ*m (d). Every experiment was repeated at least three times; error bars indicate mean ± SD (^∗^*P* < 0.05; ^∗∗^*P* < 0.01; ^∗∗∗^*P* < 0.001).

To confirm that MVs influence autophagic activity in HL-1 cells after H_2_O_2_ stimulation, we compared these cells with the H_2_O_2_-only group. The expression of Beclin-1 and LC3-II was found to be significantly enhanced, and the expression of P62 was significantly decreased in the H_2_O_2_+MVs group (Figures 4(a) and 4(b)), whereas the levels of LC3-II, Beclin-1, and P62 were higher in the H_2_O_2_+500 *μ*g-MVs group compared with the H_2_O_2_+200 *μ*g-MVs group (Figures 4(a) and 4(b)). Compared with the H_2_O_2_-treated group, both autophagosome markers were significantly increased in the MVs-treated group. The autophagosomes detected by TEM were similar to those detected by mRFP-GFP-LC3 double fluorescence (Figures 4(c) and 4(d)). Therefore, increased cell autophagy induced by hESC-NSC-derived MVs might serve as an important mechanism responsible for their cytoprotective effects after H_2_O_2_ stimulation.

### 3.4. Transfer of HSP-70 between Cells via hESC-NSC-Derived MVs

**Figure 5:**
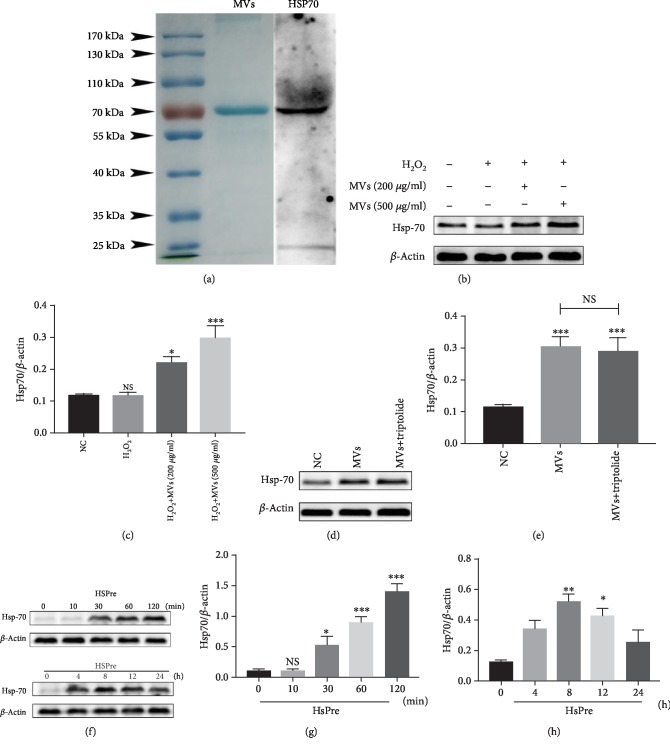
Transfer of HSP-70 between cells via hESC-NSCs-derived MVs. Coomassie blue staining and western blot analysis of protein extracts from MVs (a). Western blot images showing the protein levels of the HSP-70 after HSPre with/without triptolide pretreatment (b–e). Western blot images showing the protein levels of the HSP-70 in HL-1 cells were exposed to 42°C for 10, 30, 60, and 120 min and then allowed to recover at 37°C for 0, 4, 8, 12, and 24 h. *β*-Actin was used as an internal control (f–h). Every experiment was repeated at least three times. Error bars indicate mean ± SD (^∗^*P* < 0.05; ^∗∗^*P* < 0.01; ^∗∗∗^*P* < 0.001).

To explore the mechanism of hESC-NSC-derived MVs action in HL-1 cells, we determined which proteins were present in the MVs by SDS-PAGE analysis. Coomassie blue staining showed that the protein extract from hESC-NSC-derived MVs was mainly composed of a protein with a molecular weight of 70 kDa. We speculated that this protein was HSP-70, and HSP-70 was identified by western blot analysis (Figure 5(a)). To confirm whether HSP-70 was upregulated in HL-1 cells by MVs, the expression levels of HSP-70 in MVs-treated HL-1 cells were measured by western blotting. The results showed that HSP-70 was significantly upregulated in HL-1 cells under MV treatment and was positively correlated with the concentration of MVs (Figures 5(b) and 5(c)). In addition, the HSP-70 inhibitor triptolide (100 nM) did not reverse this change (Figures 5(d) and 5(e)). These data indicated that MVs possibly attenuate cell apoptosis under oxidative stress by transferring HSP-70. We determined that HL-1 apoptosis was affected by HSP-70 via increasing intracellular HSP-70 levels through heat shock preconditioning [23]. HL-1 cells were exposed to a temperature of 42°C for 10, 30, 60, or 120 min and then allowed to recover at 37°C for 0, 4, 8, 12, or 24 h, after which HSP-70 expression was determined. HSP-70 expression was significantly higher in the group of HL-1 cells that were exposed to 42°C for 30 min and then allowed to recover for 8 h (Figures 5(f)–5(h)). Therefore, in the following experiments, HL-1 cells that recovered for 8 h after the termination of preconditioning at 42°C for 30 min were chosen for heat shock preconditioning.

### 3.5. Antiapoptotic Effects of HSP-70 In Vitro

**Figure 6:**
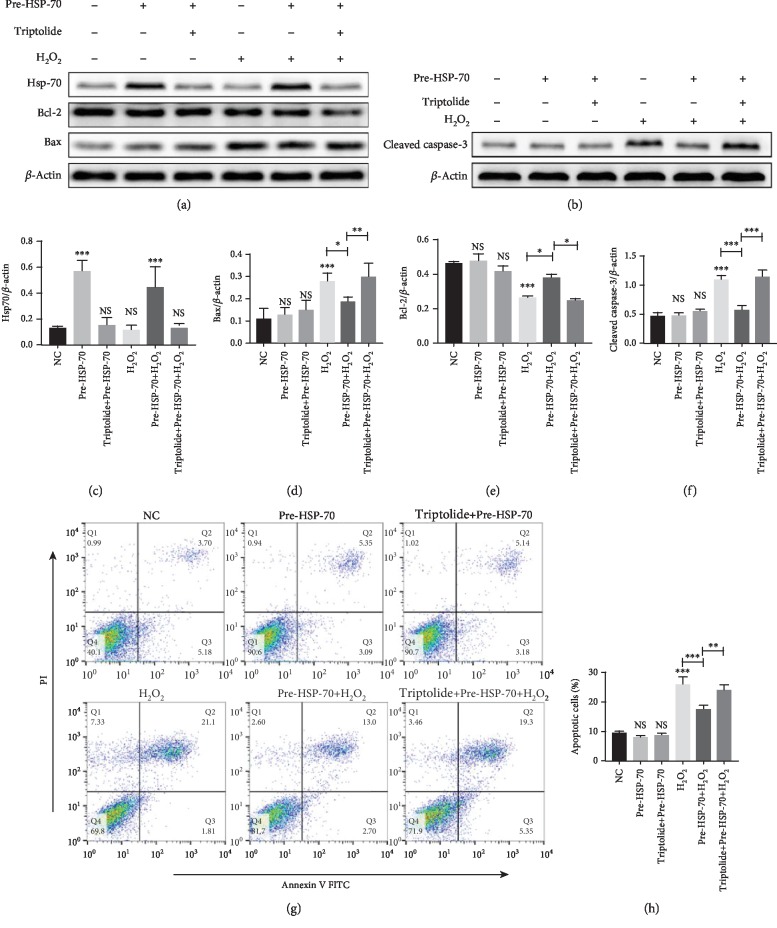
Antiapoptotic effects of HSP-70 in vitro. (a–f) Representative western blot images showing the protein levels of HSP-70, Bcl-2, Bax, and cleaved caspase-3. *β*-Actin was used as an internal control. (g) Representative dot plots of cell apoptosis were showed after Annexin V/PI dual staining. (h) The percentage of apoptotic cells was represented for both early and late apoptotic cells. Every experiment was repeated at least three times. Error bars indicate mean ± SD (^∗^*P* < 0.05; ^∗∗^*P* < 0.01; ^∗∗∗^*P* < 0.001).

Western blotting analyses performed at the end of H_2_O_2_ stimulation of HL-1 cells that had received heat preconditioning revealed significantly higher levels of HSP-70 than in the nonheated controls. Inhibition of HSP-70 by triptolide preconditioning significantly attenuated the heat preconditioning-induced overexpression of HSP-70 (Figures 6(a) and 6(c)). To detect the effects of HSP-70 on H_2_O_2_-induced apoptosis in HL-1 cells, we measured the percentage of apoptotic cells in six experimental groups of HL-1 cells using flow cytometry. As shown in (Figures 6(g) and 6(h)), there were no significant differences between the normothermic controls and the triptolide-preconditioned and HSPre-treated cells in terms of apoptosis, but the differences between the H_2_O_2_-treated cells and the control and HSPre cells were all significant, and triptolide preconditioning significantly attenuated the benefits of HSPre. In line with the above findings, there were no significant differences between the normothermic controls and the triptolide-preconditioned and HSPre-treated cells in terms of the levels of Bax, cleaved caspase-3, and Bcl-2. HSPre significantly reduced the level of Bax and cleaved caspase-3 and upregulated the level of Bcl-2 in H_2_O_2_-induced cells (Figures 6(a)–6(f)). Again, triptolide preconditioning (60 min before HSPre) significantly attenuated the benefits of HSPre.

### 3.6. HSP-70 Increased the Level of Cell Autophagy after H_2_O_2_ Stimulation

**Figure 7:**
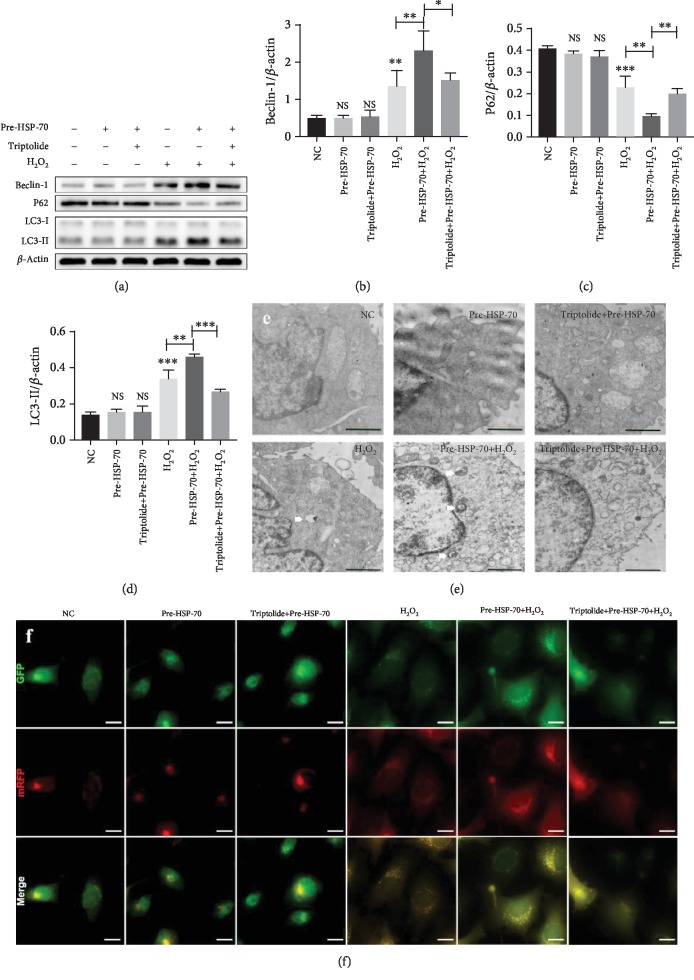
HSP-70 increased the level of cell autophagy after H_2_O_2_ stimulation. (a–d) Representative western blot images showing the protein levels of LC3, Beclin-1, and P62. *β*-Actin was used as an internal control. (e) Autophagosomes (white arrow) were detected by tandem fluorescent mRFG-GFP-LC3 assay, scale bars: 15 *μ*m. (f) Autophagosomes were measured by TEM in four groups. Every experiment was repeated at least three times, scale bars: 2 *μ*m. Error bars indicate mean ± SD (^∗^*P* < 0.05; ^∗∗^*P* < 0.01; ^∗∗∗^*P* < 0.001).

To confirm that HSP-70 influenced autophagic activity in HL-1 cells after H_2_O_2_ stimulation, we compared these cells with the H_2_O_2_-only group. The expression of Beclin-1 and LC3-II was significantly enhanced, and the expression of P62 was significantly decreased in the H_2_O_2_+HSP-70 group. In contrast, inhibition of HSP-70 by triptolide significantly attenuated the benefits of HSPre (Figures 7(a)–7(d)). There were no significant differences between the normothermic controls and the triptolide-preconditioned and HSPre-treated cells in the levels of P62 Beclin-1 and LC3B-II (Figures 7(a)–7(d)). Similar results were obtained for autophagosomes detected by TEM and mRFP-GFP-LC3 double fluorescence (Figures 7(e) and 7(f)).

### 3.7. hESC-NSC-Derived MVs Regulate Akt and mTOR Pathways by Transporting HSP-70 after H_2_O_2_ Stimulation

**Figure 8:**
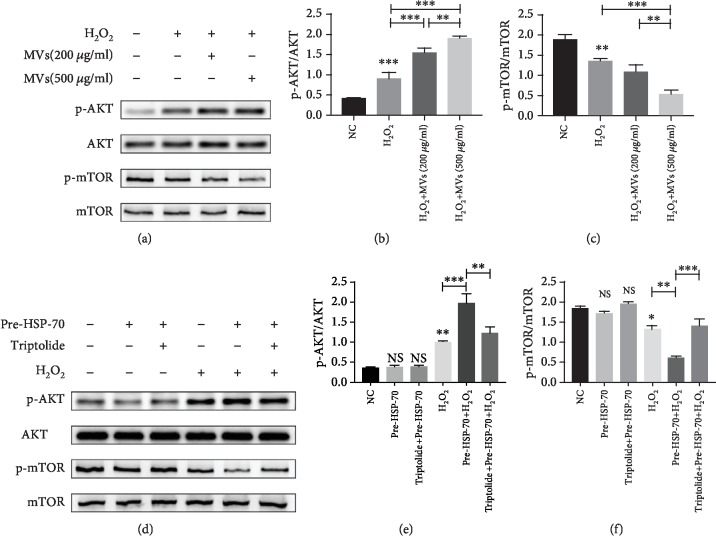
hESC-NSC-derived MVs regulate Akt and mTOR pathways by transporting HSP-70 after H_2_O_2_ stimulation. (a–f) Representative western blot images showing the protein levels of AKT, p-AKT, mTOR, and p-mTOR. *β*-Actin was used as an internal control. Every experiment was repeated at least three times. Error bars indicate mean ± SD (^∗^*P* < 0.05; ^∗∗^*P* < 0.01; ^∗∗∗^*P* < 0.001).

Cell apoptosis and autophagy are known to be associated with many signalling pathways, and the involvement of Akt and mTOR signalling has been documented in MRI [24, 25]. To investigate how hESC-NSC-derived MVs protected against H_2_O_2_-induced apoptosis in HL-1 cells, we first determined whether Akt and mTOR could be activated by hESC-NSC-derived MVs. In HL-1 cells, we found that the phosphorylation of Akt was significantly enhanced and phosphorylation of mTOR was significantly reduced after H_2_O_2_ treatment. hESC-NSC-derived MVs significantly creased the phosphorylation of Akt and reduced phosphorylation of mTOR (Figures 8(a)–8(c)). Additionally, compared to the H_2_O_2_ group, HSP-70 pretreatment by heat preconditioning significantly creased the phosphorylation of Akt and reduced phosphorylation of mTOR in HL-1 cells, which could be significantly reversed by triptolide (Figures 8(d)–8(f)). There were no significant differences between the normothermic controls and the triptolide-preconditioned and HSPre-treated cells in the level of p-AKT/AKT and p-mTOR/mTOR (Figures 8(d)–8(f)).

## 4. Discussion

Despite significant advances in the treatment of cardiovascular disease, ischaemic heart disease remains one of the leading causes of death worldwide. Stem cell-based therapies have shown promise in the repair of harmful myocardial remodelling and cardiac dysfunction, but there are still major obstacles to this approach. In this context, the amplification and delivery of beneficial paracrine signals produced by stem cells can overcome the barriers associated with cell-based injection-based methods for repairing the damaged myocardium [26].

Many studies have demonstrated that MVs serve as a mediator regulating cell-cell communication, such as that between stromal cells and breast cancer cells or mesenchymal stem cells and endothelial cells. The understanding of MV biogenesis and endocytosis is incomplete, and whether MVs may specifically recognize their receptor cells still needs to be deeply explored [27]. In MV biogenesis, plasma membrane proteins and molecules existing in the cytoplasm are actively selected in MV compartments through mechanisms that are not fully understood. Most components enriched in the MV compartment are molecules that are common to all MVs, irrespective of the origin of their producer cells, while a small set of enriched cellular components are cell-type specific molecules reflecting the nature and pathophysiological states of the specific producer cells. These enriched producer cell-specific molecules have different functions [8].

In this study, we used H_2_O_2_ to induce oxidative stress to mimic the microenvironment of cardiomyocytes in certain cardiovascular diseases. The results demonstrated that hESC-NSC-derived MVs enhanced autophagy and reduced cell apoptosis in HL-1 cells stimulated by H_2_O_2_. By using flow cytometry analysis and detection of the protein levels of Bcl2 and Bax, it was shown that H_2_O_2_-induced apoptosis in HL-1 cells can be attenuated by hESC-NSC-derived MVs. Simultaneously, the detection of autophagosomes by LC3-GFP-RFP and TEM analyses of the expression levels of the autophagy-associated proteins P62, Beclin-1, and LC3II demonstrated that hESC-NSC-derived MVs enhance H_2_O_2_-induced autophagy in HL-1 cells. Our experiments also demonstrated that these effects are partially mediated through Akt signalling. To explore the mechanism of hESC-NSC-derived MV action in HL-1 cells, we determined which proteins are contained in MVs by using SDS-PAGE analysis. Interestingly, we found that the protein extract from hESC-NSC-derived MVs was mainly composed of HSP-70. In the present study, we labelled hESC-NSC-derived MVs with Dil and incubated them with HL-1 cells. Not only were HL-1 cells able to take up MVs, but HSP-70 from MVs could also enter the cells. The expression levels of HSP-70 in HL-1 cells were significantly increased after preincubation with the hESC-NSC-derived MVs, and the HSP-70 inhibitor triptolide did not reverse this change. Then, we determined that HL-1 apoptosis was affected by HSP-70 via increasing intracellular HSP-70 levels by heat shock preconditioning. Although HSPre treatment of cells that were not treated with H_2_O_2_ did not influence apoptosis and autophagy, HSPre caused HSP-70 overexpression and significantly enhanced autophagy and reduced cell apoptosis in HL-1 cells. The beneficial effects of HSPre on apoptosis and autophagy were significantly attenuated by triptolide preconditioning, which inhibited HSP-70 overexpression. These observations prompted us to hypothesize that HSP-70 attenuates H_2_O_2_-stimulated HL-1 myocyte death by inhibiting apoptosis and enhancing autophagy.

The heat shock response, a transcriptional response that upregulates molecular chaperones upon heat shock, is necessary for cell survival in a stressful environment to maintain protein homeostasis (proteostasis) [28]. HSP-70 is a highly conserved and ubiquitous protein that maintains cell homeostasis as well as homeostasis throughout the organism. Under normal conditions, HSP-70 predominantly resides in the cytosolic compartment, where it supports the folding, refolding, and assembly of nascent polypeptides, prevents protein aggregation, and assists in the transport of other proteins across membranes [29]. Following nutritional deprivation, chemical stress, or physical interventions, such as exposure to ionizing radiation, hypoxia, or hyperthermia, the synthesis of HSP-70 is faster than that of other stress proteins, and HSP-70 accumulates at higher levels after stress to maintain cell homeostasis and homeostasis of the entire organism [28]. It has been reported that HSP-70 is secreted from cells and is mainly transmitted between cells by MVs [28, 30, 31]. Moreover, HSP-70 has long been regarded as a specific marker of MVs [32].

Here, we investigated the biological effects of exogenous HSP-70 transported by hESC-NSC-derived MVs on cardiomyocyte survival. Our experiments suggest that hESC-NSC-derived MVs rescue cardiomyocyte apoptosis by transporting HSP-70 to induce autophagy in cardiomyocytes. Of course, this hypothesis requires further validation in animal experiments.

## 5. Conclusions

In summary, our experimental results show that hESC-NSC-derived MVs inhibit the apoptosis of HL-1 cardiomyocytes. Moreover, we showed that MVs may exert their effect by promoting autophagy and regulating AKT and mTOR pathways via transporting HSP-70. Overall, the application of hESC-NSC-derived MVs might be a potential therapeutic strategy for IHD.

## Data Availability

The data that support the findings of this study are available from the corresponding author upon reasonable request.

## Supplementary Materials

Supplementary Materials Supplementary Figure S1: supplementary identification of NSC.
