# Supplementary material for: Microvesicles Derived from Human Embryonic Neural Stem Cells Inhibit the Apoptosis of HL-1 Cardiomyocytes by Promoting Autophagy and Regulating AKT and mTOR via Transporting HSP-70
Source: Stem Cells Int. 2019 Oct 24;2019:6452684. doi: 10.1155/2019/6452684 (PMC6854932; doi:10.1155/2019/6452684)
Supplement: Supplementary Materials — Supplementary Figure S1: supplementary identification of NSC. [file 6452684.f1.pdf]

Supplementary Figure S1

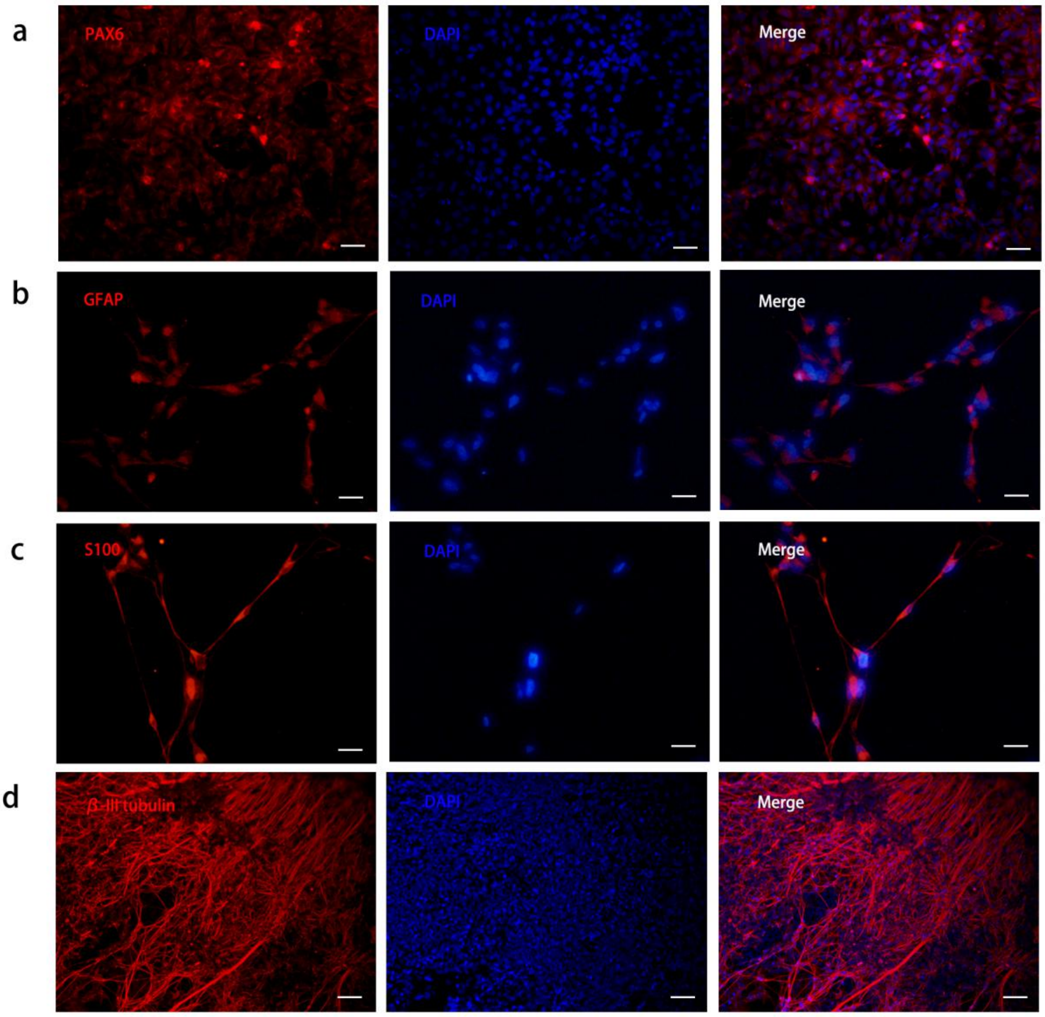

Supplementary Figure S1: Supplementary identification of NSC. hESC-NSC were immunofluorescence staining for pax6 (a). Immunofluorescence staining for GFAP, S100 and β-III tubulin of glial cells and neurons directly differentiated by hESC-NSC (b-d). (a-d: Scale bars 50 μm).
